# Long-term host–pathogen evolution of endogenous beta- and gammaretroviruses in mouse lemurs with little evidence of recent retroviral introgression

**DOI:** 10.1093/ve/veac117

**Published:** 2022-12-14

**Authors:** Sharon E Kessler, Kyriakos Tsangaras, Solofonirina Rasoloharijaona, Ute Radespiel, Alex D Greenwood

**Affiliations:** Faculty of Natural Sciences, University of Stirling, Stirling FK9 4LA, Scotland; Department of Wildlife Diseases, Leibniz Institute for Zoo and Wildlife Research (IZW), Alfred-Kowalke-Straße 17, Berlin 10315, Germany; Department of Wildlife Diseases, Leibniz Institute for Zoo and Wildlife Research (IZW), Alfred-Kowalke-Straße 17, Berlin 10315, Germany; Department of Life and Health Sciences, University of Nicosia, 46 Makedonitissas Avenue, CY-2417, P.O. Box 24005, Nicosia, CY-1700, Cyprus; Faculty of Science, Technology and Environment, University of Mahajanga, 5 Georges V Street - Building KAKAL Mahajanga Be - Po. Box 652 , Mahajanga 401, Madagascar; Institute of Zoology, University of Veterinary Medicine Hannover, Foundation, Buenteweg 17, Hannover 30559, Germany; Department of Wildlife Diseases, Leibniz Institute for Zoo and Wildlife Research (IZW), Alfred-Kowalke-Straße 17, Berlin 10315, Germany; Department of Veterinary Medicine, Freie Universität Berlin, Oertzenweg 19 b, Berlin 14163, Germany

**Keywords:** endogenous retrovirus, lemur evolution, betaretrovirus, gammaretrovirus, *Microcebus*, Madagascar

## Abstract

Madagascar’s flora and fauna have evolved in relative isolation since the island split from the African and Indian continents. When the last common ancestors of lemurs left Africa between 40 and 70 million years ago, they carried a subset of the viral diversity of the mainland population within them, which continued to evolve throughout the lemur radiation. Relative to other primate radiations, we know very little about the past or present viral diversity of lemurs, particularly mouse lemurs. Using high-throughput sequencing, we identified two gammaretroviruses and three betaretroviruses in the genomes of four species of wild mouse lemurs. The two gammaretroviruses and two betaretroviruses have not previously been described. One betaretrovirus was previously identified. All identified viruses are present in both Lorisiformes and Lemuriformes but absent from haplorrhine primates. The estimated ages of these viruses are consistent with the estimated divergence dates of the host lineages, suggesting they colonized the lemur genome after the Haplorrhine–Strepsirrhine split, but before the Lorisiformes–Lemuriformes split and before the colonization of Madagascar. The viral phylogenies connect multiple lineages of retroviruses from non-lemur and non-Madagascar-native species, suggesting substantial cross-species transmission occurred deep in the primate clade prior to its geographic dispersal. These phylogenies provide novel insights into known retroviral clades. They suggest that the origin of gammaretroviruses in rodents or bats may be premature and that the Jaagsiekte sheep virus clade may be older and more broadly distributed among mammals than previously thought.

## Introduction

When the island of Madagascar broke away from Mainland Africa 165–121 mya (Rabinowitz, Coffin, and Falvey 1983; Roos, Schmitz, and Zischler 2004) and then from India ∼88 mya (Storey et al. 1995; Roos, Schmitz, and Zischler 2004), it created a natural laboratory (Yoder and Yang 2004) for understanding the processes of evolution. Since then, flora and fauna have evolved largely in isolation, punctuated by intermittent invasions from Africa and Asia. The first primates on Madagascar were one of those invasions; they arrived in Madagascar between 40 and 70 mya (Roos, Schmitz, and Zischler 2004; Yoder and Yang 2004; Poux et al. 2005; Perelman et al. 2011; Kistler et al. 2015; Herrera and Davalos 2016), possibly by rafting across the channel (Kappeler 2000; Roos, Schmitz, and Zischler 2004; Poux et al. 2005; Ali and Huber 2010; Nowack and Dausmann 2015; Dausmann and Warnecke 2016; Mazza, Buccianti, and Savorelli 2019; Masters et al. 2021). They subsequently radiated to fill the diverse niches that are typically occupied by monkeys and apes in Africa and Asia, making the lemur radiation on Madagascar a natural comparison with the radiations that occurred in other branches of the primate tree elsewhere.

When the last common ancestors of lemurs left Africa, they carried a subset of the viral diversity of the mainland population within them, which continued to evolve as their hosts underwent the lemur radiation as we know it today. Viruses generally evolve far more rapidly than their hosts, making current viral diversity frequently very different from past viral diversity (Gifford 2012; Greenwood et al. 2018). This discordance in evolutionary rates makes it difficult to reconstruct past viral diversity and to understand how current viruses evolved from those in the past.

Endogenized viruses, which have been ‘fossilized’ in the host genome (Feschotte and Gilbert 2012; Gifford 2012; Grandi and Tramontano 2018; Greenwood et al. 2018; Katsura and Asai 2019; Geis and Goff 2020; Luganini and Gribaudo 2020; Xue, Sechi, and Kelvin 2020; Arneth 2021; Ferrari et al. 2021; Grandi et al. 2021), can help to fill in the gaps. Typically, viruses are transmitted horizontally among individuals (Feschotte and Gilbert 2012; Gifford 2012; Grandi and Tramontano 2018; Greenwood et al. 2018; Katsura and Asai 2019; Geis and Goff 2020; Luganini and Gribaudo 2020; Xue, Sechi, and Kelvin 2020; Arneth 2021; Ferrari et al. 2021; Grandi et al. 2021). However, if the virus invades the germline cells of the host, it becomes ‘endogenized’ and is transmitted from generation to generation as a Mendelian trait (Feschotte and Gilbert 2012; Gifford 2012; Grandi and Tramontano 2018; Greenwood et al. 2018; Katsura and Asai 2019; Geis and Goff 2020; Luganini and Gribaudo 2020; Xue, Sechi, and Kelvin 2020; Arneth 2021; Ferrari et al. 2021; Grandi et al. 2021). Endogenized viral elements are particularly informative for learning about the evolutionary history of viruses because they no longer mutate at the high rate typical of exogenous viruses, thus ‘fossilizing’ the genetic structure of the ancient virus within the host’s genome. Moreover, endogenous viruses may persist in the host genome long after the original exogenous species have become extinct, providing insight into past viral diversity and becoming involved in complex host–virus evolutionary dynamics (Feschotte and Gilbert 2012; Gifford 2012; Grandi and Tramontano 2018; Greenwood et al. 2018; Katsura and Asai 2019; Geis and Goff 2020; Luganini and Gribaudo 2020; Xue, Sechi, and Kelvin 2020; Arneth 2021; Ferrari et al. 2021; Grandi et al. 2021).

Compared with other primate radiations (e.g. Catarrhines) (Switzer et al. 2005; Stengel et al. 2006; Liu et al. 2008; Locatelli and Peeters 2012; Jasinska et al. 2013), very little is known about either present or past viral diversity in lemurs. This reduces our insight into how primates and viruses have co-evolved and how it limits our ability to monitor and predict cross-species transmissions, including with humans. Large-scale studies of current, exogenous viruses in lemurs are rare but suggest that significant undescribed viral diversity is circulating among lemur populations (Zohdy et al. 2015). Even less is known about endogenous viral diversity in lemurs, but it has offered some surprising insights into the evolutionary history of some of the more extensively researched viral groups (Gifford 2012). For example, non-human primate simian immunodeficiency viruses (SIVs) (lentiviruses related to human immunodeficiency virus) were thought to occur only in Catarrhines until the recent discovery of endogenous prosimian SIV in mouse lemurs (*Microcebus* ssp.) and fat-tailed dwarf lemurs (*Cheirogaleus medius*) (Gifford et al. 2008; Gilbert et al. 2009; Rahm et al. 2011; Gifford 2012). An additional endogenized virus, a betaretrovirus, has been detected in unordered bacterial artificial sequences from the gray mouse lemur, possibly a result of having been infected by a rodent host (Baillie et al. 2004). The sequence identified contains frameshifts and premature termination mutations in the *gag* and *pro* open reading frames and deletions in the 3ʹ end of the *pol* gene and the 5ʹ end of the *env* gene. The *pol* gene also contains premature stop codons (Baillie et al. 2004).

We focused on the mouse lemurs (*Microcebus* spp.). Several lines of evidence suggest that mouse lemurs may be particularly informative for understanding viral evolution in lemurs, including as sentinel species (Zohdy et al. 2015), for detecting cross-species spillover events. They are the smallest-bodied species of the lemurs (30–80 g), have small home ranges, subsist on an omnivorous diet, and are flexible in their use of torpor (Dausmann and Warnecke 2016; Radespiel 2016; Yoder et al. 2016; Ezran et al. 2017). This enables them to survive in diverse forests across Madagascar (Radespiel 2016; Yoder et al. 2016; Ezran et al. 2017), including in small, disturbed fragments or edge habitats that are unsustainable for larger species (Ganzhorn and Schmid 1998; Andriamandimbiarisoa et al. 2015; Setash et al. 2017; Knoop, Chikhi, and Salmona 2018; Schuessler et al. 2018; Steffens and Lehman 2018; Andriatsitohaina et al. 2019; Montero et al. 2019). As a result, they are likely to be sympatric with other wildlife in larger forest patches and with domestic animals and humans in more disturbed habitats, making them a potential link for cross-species transmissions (Zohdy et al. 2015). Moreover, because they are small, short-lived, rapidly reproducing mammals (Radespiel et al. 2019) capable of living at high densities (Setash et al. 2017), they may be able to endure higher viral loads due to reduced selection against tumors (for a review of tumors in prosimians, including mouse lemurs, see Remick, Van Wettere, and Williams 2009), similar to what has been hypothesized for why rodents and bats may be common links in cross-species retrovirus transmissions (Greenwood et al. 2018)).

There are currently twenty-four recognized species of mouse lemurs, which exist in complex patterns of sympatry, allopatry, and hybridization zones across the Malagasy forests (Radespiel 2016; Yoder et al. 2016; Poelstra et al. 2020). The age estimates for the basal diversification vary substantially; fossil-calibrated estimates produced divergence times of  9–10 mya (Yang, Yoder, and Thorne 2003; Yoder and Yang 2004; Yoder et al. 2016; Dos Reis et al. 2018), while newer pedigree-based methods (Campbell et al. 2021) produced an estimate of ∼1.5 mya (Poelstra et al. 2020). The mouse lemur clade has three major lineages, and this study includes species from each of them: (1) *Microcebus murinus*, (2) *Microcebus ravelobensis* and *Microcebus bongolavensis*, and (3) *Microcebus myoxinus* (Yoder et al. 2000, 2016; Heckman et al. 2007; Weisrock et al. 2010, 2012). The International Union for Conservation of Nature (IUCN) reports each of these species as having decreasing populations and classifies *M. murinus* as least concern, *M. ravelobensis* as vulnerable, *M. bongolavensis* as endangered, and *M. myoxinus* as vulnerable (IUCN 2021).

Applying high-throughput sequencing (HTS) to these four species of mouse lemurs from wild populations sampled in northwestern Madagascar, we have identified multiple broadly distributed mouse lemur gamma- and betaretroviruses. They suggest substantial cross-species transmissions from multiple lineages of retroviruses from non-lemur and non-Madagascar-native species occurred prior to the primate colonization of Madagascar with subsequent host and viral evolution.

## Methods

### Sample collection

**Figure 1.**
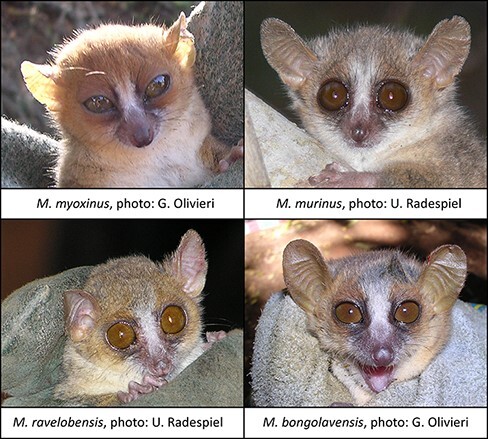
Photos of the four studied mouse lemur species: *M. myoxinus*, photo: G. Olivieri, 2003; *M. murinus*, photo: U. Radespiel, 2013; *M. ravelobensis*, photo: U. Radespiel, 2004; *M. bongolavensis*, photo: G. Olivieri, 2003.

Four species of wild mouse lemurs (Fig. 1) were trapped in Madagascar from August to September 2013 at four sites in the northwest (Table 1). All sites are dry deciduous forests, which experience a cool, dry season (May–October) and a hot, humid, rainy season (November–April). All sites are under heavy anthropogenic pressure. Jardin Botanique A and B (JBA and JBB) are in Ankarafantsika National Park. The forest in Mahatazana (16°08ʹ S, 47°41ʹ E) is near the Mahajamba River and borders rice fields. The Bombetoka site (15°52ʹ S, 46°15ʹ E) is approximately 20 km south of the city of Mahajanga at the western bank of the Betsiboka River. It is highly fragmented with clear signs of cattle-grazing and slash and burn clearing.

**Table 1.** Study species and sample sizes, broken down by sex and field site.

Species	M	F	Site
*M. bongolavensis*	4	6	Mahatazana
*M. murinus*	6	4	Ankarafantsika, JBA
*M. murinus*	1	3	Bombetoka
*M. myoxinus*	3	7	Bombetoka
*M. ravelobensis*	2	3	Ankarafantsika, JBA
*M. ravelobensis*	5	5	Ankarafantsika, JBB

Ankarafantsika sites JBA and JBB are only a few kilometers apart, thus the *M. ravelobensis* at these sites are considered to belong to one population.

Lemurs were caught in Sherman Live traps, which were baited with banana, at dusk and collected at dawn using published procedures (Radespiel 2000; Radespiel et al. 2001). As mouse lemurs are seasonal breeders, females did not have dependent young at the time of sampling. Following veterinary protocols of the University of Veterinary Medicine Hannover, we carefully drew blood from either the femoral or saphenous vein on the inside of each lemur’s thigh on both legs with a sterile, single-use micro-lancet and collected the drop of blood that welled up with a single-use plastic transfer micropipette. Approximately, 25 µl of whole blood per individual was preserved in RNAlater (Qiagen). Samples were frozen within several days (most within hours). The lemurs were given more banana and released at their capture sites on the same evening that blood was drawn. We collected blood samples from twenty-three* M. bongolavensis,* thirty-three* M. murinus,* nine *M. myoxinus,* and forty-two * M. ravelobensis* and selected a total of forty-nine samples for this study (Table 1).

Laboratory analysis was carried out in the Department of Wildlife Diseases at the Leibniz Institute for Zoo and Wildlife Research in Berlin, Germany. Methods were approved by the Malagasy Government and by Madagascar National Parks (field permits granted to the University of Veterinary Medicine Hannover: 156/13/MEF/SG/DGF/DCB.SAP/SCB; 168/13/MEF/SG/DGF /DCB.SAP/SCB; and 169/13/MEF/SG/DGF/DCB.SAP/SCB).

### Extraction and reverse transcription

RNA was extracted from blood using the protocol for purification of viral nucleic acids from plasma or serum (QiAamp Min Elute Virus Spin Kit, Qiagen, Germany). We modified the protocol by using 20 µl of proteinase K (Invitrogen, Germany) instead of the Qiagen protease that was provided, followed by the addition of 5 µl of linear acrylamide (5 mg/ml, Invitrogen, Germany). A DNase step was included in the AW1 buffer step. Samples were spun down using half of the recommended AW1 buffer, then added 10 µl ×10 TURBO DNase buffer (Ambion, Germany) and 1 µl of TURBO DNase (Ambion, Germany), and incubated it at 37°C for 15 min before applying the second half of the AW1 buffer and centrifuging. During the final elution step, samples were incubated for 5 min before centrifugation.

RNA was reverse transcribed into double-stranded cDNA in duplicate for each sample. Eleven microliters of each sample was mixed with 1 µl of Random Hexamers (50 µM, Invitrogen, Germany) and incubated at 65°C for 5 min and then for 2 min on ice. Four microliters of 5X Superscript III Buffer (Invitrogen, Germany) was combined with 1 µl of 10 mM dNTPs (New England BioLabs, Ipswich, USA), 1 µl of 0.1 M DTT, 1 µl of RNaseOUT Recombinant Ribonuclease Inhibitor (Invitrogen, Germany), and 1 µl Superscript III Reverse Transcriptase (Invitrogen, Germany). The samples were incubated at 25°C for 10 min, 50°C for 50 min, 85°C for 5 min, then put on ice for 2 min, incubated at 95°C for 2 min, and put on ice for 2 min. Second strand synthesis was done by adding 1 µl of DNA Polymerase I Klenow (5000 U/ml, New England BioLabs) and incubating at 37°C for 1 h and then at 75°C for 20 min. Double-stranded cDNA was quantified using the Qubit dsDNA HS assay (ThermoFisher Scientific, MA, USA) yielding concentrations ranging from 0.324 to 10.1 ng/µL.

### DNA shearing, library building, and sequencing

Samples were sheared to a target length of 400 bp using a Covaris M220 Focused-Ultrasonicator in 50 or 15 µl tubes. When using a 50 µl tube, 20 µl of cDNA and 30 µl of water were used, following the manufacturer’s instructions for obtaining a target length of 400 bp. When using a 15 µl tube, 15 µl of cDNA was used, and a treatment time of 37 s was applied. After shearing, samples were purified using a QIAquick PCR Purification Kit (Qiagen, Germany).

Illumina libraries were produced based on published methods (Meyer and Kircher 2010) as follows. First, an oligo hybridization buffer (×10) with a pH of 8.0 was made using 29.22 g/l 500 mM NaCl, 1.57 g/l 10 mM Tris–Cl (pH 8.0), and 0.292 g/l 1 mM EDTA (pH 8.0). A hybridization mix for adapter P5 was produced and diluted to a final concentrations of 200 µM by mixing 20 µl IS1_adapter_P5.F (500 µM), 20 µl IS3_adapter_P5 + P7.R (500 µM), 5 µlo hybridization buffer (×10), and 5 µl water. A hybridization mix for adapter P7 was produced and diluted to a final concentration of 200 µM by mixing 20 µL IS2_adapter_P7.F (500 µM), 20 µL IS3_adapter_P5 + P7.R (500 µM), 5 µl oligo hybridization buffer (×10), and 5 µl water. Reactions were incubated for 10 s at 95°C, followed by a ramp from 95°C to 12°C at a rate of 0.1°C/s. We combined both reactions to produce an adapter mix with a concentration of 100 µM for each adapter.

Double-stranded DNA samples were built into Illumina libraries using the following three steps: (1) blunt end reaction was performed using the NEBNext End Repair module (E6050; New England BioLabs) following the manufacturer’s instructions. Blunt end samples were then purified using a QIAquick PCR Purification Kit and eluted at 32 µl. (2) Illumina adapter ligation was performed on the repaired samples using NEBNext Quick Ligation module (E6056; New England BioLabs) following the manufacturer’s instructions. Adapter-ligated products were purified using a QIAquick PCR Purification Kit. Reactions were eluted with 42 µl buffer EB and the column was incubated at 37°C for 5 min in a heat block before spinning down the DNA at 13,000 rpm for 1 min. (3) Adapter fill in was performed to remove nicks from the adapters using Bst Polymerase Kit (M0275S; New England BioLabs) as follows: mixing 40 µl of DNA eluted from Step 2, 5 µl of ThermoPol Reaction Buffer, 2 µl of 10 mM dNTPs (New England BioLabs), and 3 µl of Bst DNA polymerase, which was incubated in a thermal cycler for 20 min at 65°C and 20 min at 80°C. Adapter-ligated products were double indexed using the Herculase II Fusion DNA Polymerase (Agilent, Germany) in 50 µl reactions containing 10 µl Herculase Buffer, 0.5 µl dNTPs (25 mM, Agilent, Germany), 1 µl P5, 1 µl P7, 5–6.3 µl template library, 0.5 µl Herculase Polymerase, and water up to 50 µl. Amplification of the libraries was performed using the following cycling conditions: 95°C for 5 min, 5–23 cycles of 95°C for 30 s, 60°C for 30 s, and 72°C for 40 s, and a final 7 min elongation at 72°C. Illumina libraries were purified with a QIAquick PCR Purification Kit. Following purification, the forty-nine libraries were grouped into four pools of 10–15 libraries each. Size selection was performed on the pools for 250–600 bp fragments using a Pippin Prep Kit (Sage Science, Biozym Scientific in Olendorf, Germany). Final libraries were pooled equimolarly to a final library concentration of 4 nM for paired-end sequencing (2×300) on an Illumina MiSeq platform with the V3 reagent kit.

### HTS data viral screening

Fastq files (SRA accession no. PRJNA779192) containing the raw data for each of the forty-nine samples that had sequencing output ranging from 200 to 400 Mb were scanned for adapter sequences and quality trimmed with Cutadapt (Marcel 2011), using a quality cut-off of 30 bp and a minimum length cut-off of 30 bp. Trimmed paired-end files for each sample were merged creating a single data file that was further processed with a modified version of the Virus Identification Pipeline (VIP). VIP (Li et al. 2016) is a bioinformatic pipeline that screens next generation sequencing data for viral hits. Each sample’s fastq file was run through the pipeline using the sense mode. The pipeline first aligns the raw reads to the human genome (hg19) and filters out all reads that are mapped to the reference genome. The remaining reads are then filtered against a bacterial database to further minimize potential unspecific matches due to bacterial sequences. The curated and filtered reads were then processed and searched against a viral genome database, followed by an amino acid alignment to a viral protein database. Viral positive hits were then further processed using Geneious Prime 2020.0.3 *de novo* assembly to generate larger contigs.

All raw reads were also processed through the genome detective server to further validate and confirm viral positive hits. Genome detective (Vilsker et al. 2019) assembles viral genomes quickly and accurately using *de novo* alignments to generate viral contigs that have high combined amino acid and nucleotide scores. Both viral screening methods revealed contig matches to betaretroviral and gammaretroviral sequences from all samples.

### *Microcebus murinus* genome retroviral screening and retroviral mining

Genome detective and VIP gamma and betaretroviral-positive contigs with an average size of 800 bp and read depth ranging from ×10 to ×300 were blast searched against the *M. murinus* genome v3.0 (Supplementary Fig. S1). Positive hit coordinates were extracted from the genomic sequence ±10,000 bp. The extracted genomic sequences were annotated using Geneious Prime and aligned using MAFFT algorithm with default settings (Katoh, Rozewicki, and Yamada 2019). Alignments were used to generate majority rule consensus sequences for the proviruses and LTRs for the corresponding retroviral hits (Supplementary fasta files). Retrotector (Sperber et al. 2007) was employed to generate putative, reconstructed protein, called puteins, for all retroviral genes on the proviral consensus. Conserved motifs were further confirmed using NCBI conserved domain database (CDD) (Marchler-Bauer et al. 2011).

### Recombination screening

Extracted scaffold proviral alignments generated were screened using the Recombination Analysis Tool (RAT) with the default settings for potential recombination events (Etherington, Dicks, and Roberts 2004).

### Genome assemblies retroviral screening

Genome assemblies obtained from NCBI were used to determine the presence of the identified betaretroviral and gammaretroviral sequences in species other than *M. murinus, M. bongolavensis, M. ravelobensis*, and *M. myoxinus*.

**Haplorrhines** (**Catarrhines**): Genome data of *Homo sapiens* (GCA_000001405.28), *Pan paniscus* (GCA_000258655.2), *Pan troglodytes* (GCA_000001515.5), *Gorilla gorilla gorilla* (GCA_000151905.3), *Nomascus leucogenys* (GCA_000146795.3), *Theropithecus gelada* (GCA_003255815.1), *Papio anubis* (GCA_000264685.2), *Mandrillus leucophaeus* (GCA_000951045.1), *Macaca mulatta* (GCA_003339765.3), *Colobus angolensis palliatus* (GCA_000951035.1), *Piliocolobus tephrosceles* (GCA_002776525.2); **Haplorrhines (Platyrrhines):***Saimiri boliviensis boliviensis* (GCA_000235385.1), *Cebus capucinus imitator* (GCA_001604975.1), *Callithrix jacchus* (GCA_002754865.1); **Haplorrhines (Tariser):***Carlito syrichta* (GCA_000164805.2); **Strepsirrhines (Lorisiformes):***Otolemur garnettii* (GCA_000181295.3), *Nycticebus coucang* (GCA_004027815.1); **Strepsirrhines (Lemuriformes):***Propithecus coquereli* (GCA_000956105.1), *Mirza zaza* (GCA_008750895.1), *Mirza coquereli* (GCA_004024645.1), *Microcebus tavaratra* (GCA_008750935.1), *Indri indri* (GCA_004363605.1), *M. ravelobensis* (GCA_008750975.1), *Microcebus griseorufus* (GCA_008750995.1), *Microcebus mittermeieri* (GCA_008750955.1). **Non-primates:***Cryptoprocta ferox* (GCA_004023885.1), and *Microgale talazaci* (GCA_004026705.1) were obtained. Consensus retroviral sequences were used to search the above-mentioned genomes using blastn with default parameters.

### Phylogenetic analysis

Multiple alignments were generated using MAFFT v 7 with an iterative refinement method for both betaretroviral and gammaretroviral nucleotide and amino acid sequences (Katoh, Rozewicki, and Yamada 2019). Further curation was performed on both alignments as needed. Additional betaretroviral and gammaretroviral sequences were obtained from GenBank with the following accession numbers: reticuloendotheliosis virus (REV) (MF185397), *Rhinolophus ferrumequinum* retrovirus (RfRV) (JQ303225), FeLV (NC_001940), FMLV (Z11128), FMLV (D88386), MMLV (AF033811), MLV (MLMCG), MLV (AY818896), XMRV (JF908815), XMRV (JF908816), MLV (AB213653), MLV (MLVENVR), *Mus musculus* (AL606987), *Mus caroli* (XM021149499), *Grammomys surdaster* (XM_028761645), *M. musculus* (XR_001784239), *Mus pahari* (XM_021190222), *Rattus norvegicus* (XR_005497950), *R. norvegicus* (XM_039101019), *M. caroli* (XM_021185800), *M. musculus* (AC130672), CPERV (UGO47158), KWERV (GQ222416), PERV-A (KY484771) PERV-B (AY099324), PERV-C (HM159246), *Arvicanthis niloticus* (XM_034491546), McERV (KC460271), MDEV (AF053745), Gibbon ape leukemia virus (GaLV)-SF (KT724047), GaLV-Hall’s Island (KT724050), GaLV-brain (KT724049), GaLV-SEATO (KT724048), KoRV-KV522 (AB721500), KoRV Pci-SN265 (KF786285), KoRV Br2-1 CEETG (KC779547), WMV-SSAV (KT724051), MmGRV (MN413611), SaGRV (MN413612), FFRV1 (MK040728), HPG (MN413610), HlGRV(MN413613), *Cricetulus griseus* endogenous retrovirus (ERV) (XM_027403845), UrsusERV (Repbase reports 15 (11), 3519 (2015), SERV (STU85505), SERV (JN134185), simian type D retrovirus (SRV)-2 (AF126467), SRV-5 (LC426347), MPMV (AF033815), SIVMPCG (M12349), SRV-4 (AB920340), SRV-8 (KU605777), squirrel monkey retrovirus H (SMRV-H) (M23385), *Desmodus rotundus* ERV (DrERV) (MH648003), *Trichosurus vulpecula* retrovirus (TvERV) (AF224725), Jaagsiekte sheep retrovirus (JSRV) (MN161849), JSRV (M80216), endogenous virus JSRV (enJSRV)-11 (EF680303), enJSRV-16 (EF680300), enJSRV-15 (EF680299), HERV-K (Y18890), HERV-K-C19 (Y17833), HERV-K-C7-34 (Y17834), HERV-K-C7 (Y17832), MMTV (AF228551), PSRV1(MT787217), PyERV (AF500296), HML-1 (Repbase-HERV-K14I), HML-2 (Repbase-HERV-K), HML-3 (Repbase-HERVK9I), HML-4 (Repbase-HERVK13I), HML-5 (Repbase-HERVK22I), HML-6 (Repbase-HERVK3I), HML-7 (Repbase-HERVK11DI), HML-8 (Repbase-HERVK11I), HML-9 (Repbase-HERVKC4). Statistical selection of best fit models for the whole genome alignments and gene-specific protein alignments was performed using jModelTest2 (Darriba et al. 2012) and ProtTest3 (Darriba et al. 2011). Phylogenetic analysis of the nucleotide and protein alignments was performed with the RAxML bootstrap algorithm with 500 bootstrap replicates using the specified best fit models as determined by jmodelTest2 and ProtTest3 (Huelsenbeck and Ronquist 2001; Stamatakis 2014).

### Age estimation of retroviral sequences

To estimate the identified retroviruses integration ages into the mouse lemur genome, three methods employing a molecular clock approach were used. First, the divergence of LTR sequences for each retrovirus from its consensus LTR sequence was estimated, while excluding the hypermutable CpG sites from the analysis (Mayer et al. 2013). The sequence divergence was corrected based on the Kimura-based parameter (K2P) model (Kimura 1980). The calculated sequence divergence from the consensus and the reported mouse lemur mutation rate of 0.0017/nt/myr were used to estimate the evolutionary rates of the identified retroviral sequences (Campbell et al. 2021). As a second method of age estimation, we used the sequence divergence of each aligned proviral gene from its consensus sequence, again excluding the hypermutable CpG sites from the analysis. Divergence estimates were corrected using the K2P model as earlier, and using the mouse lemur reported mutation rate, to estimate the age of each provirus. Proviral 5ʹ LTR and 3ʹ LTR sequence divergence was used as a third way to estimate the age of the endogenous elements. 5ʹ LTR and 3ʹ LTR sequences upon integration into the genome are identical, and they acquire mutations independently through time based on the host mutation rate. For each proviral sequence identified, its age was estimated employing *T* = *D*/(2*0.0017) where *D* is the calculated divergence between the two sequences and 0.0017 is the host mutation rate.

## Results

### ERV identification in mouse lemur sequencing data

Viral screening of the cDNA sequences of all samples using the VIP and genome detective revealed the presence of multiple sequences that were homologous to the Orthoretroviridae gamma and betaretroviruses. *De novo* assembly of sequences was performed in all samples resulting in larger contig sequences revealing the presence of two gammaretroviruses, which we designated MicrocebusERV-1-1 and MicrocebusERV-1-2, and three betaretroviruses, which we designate MicrocebusERV-2-1, MicrocebusERV-2-2, MicrocebusERV-2-3 (previously identified by Baillie et al. 2004).

#### Gammaretroviruses

A blastn search of MicrocebusERV-1-1 showed an identity of 76 per cent to the Baboon ERV and MicrocebusERV-1-2 showed an identity of 75 per cent to the GaLV-brain.

#### Betaretroviruses

MicrocebusERV-2-1 showed an identity of 64 per cent to the endogenous virus Jaagsiekte sheep retrovirus (en-JSRV-14), MicrocebusERV-2-2 showed an identity of 67 per cent to the *Nannospalax gallii* ERV, MicrocebusERV-2-3 showed an identity of 66 per cent to the TvERV and an 97.1 per cent identity to AC145758, and the endogenous *Microcebus murinus* betaretrovirus previously identified in a bacterial artificial chromosome (BAC) sequence (Baillie et al. 2004) at the DNA level. Larger contigs for all five retroviruses were present in the majority of mouse lemur samples from all four species examined.

Consensus contig sequences for each retroviral genome identified were used as query sequences to perform a blastn search to the genome of *M. murinus* v3 (GCA_000165445).

Blastn searches revealed 1,446 hits for MicrocebusERV-1-1, 2,010 hits for MicrocebusERV-1-2, 457 hits for MicrocebusERV-2-1, 132 hits for MicrocebusERV-2-2, and 929 hits for MicrocebusERV-2-3 (Table 2). Coordinates of the blastn hits were used to extract the corresponding regions from the *M. murinus* v3 (GCA_000165445) chromosomes or scaffold sequences, including 10,000 bp both upstream and downstream of the blastn identified region. Using approximately 10,000 bp was sufficient to obtain full length ERV genomes. For the gammaretroviruses, despite the large number of blastn hits, the analysis revealed the presence of only 20 proviral copies of MicrocebusERV-1-1 and 109 proviral copies of MicrocebusERV-1-2. For the betaretroviruses, the search found five proviral copies of MicrocebusERV-2-1, thirty-nine proviral copies of MicrocebusERV-2-2, and 138 proviral copies of MicrocebusERV-2-3 (Table 2). The exact boundaries for each ERV locus were identified using repeatmasker using published methods (Tsangaras et al. 2015) (Supplementary fasta files). Extracted loci were further screened using the CENSOR software tool of repbase (Kohany et al. 2006). The CENSOR script compares the sequences provided with a reference database of repetitive elements and masks the homologous regions of the query sequence; at the same time, a report is generated indicating the classification of the matched repeats. Analysis of the extracted loci using CENSOR (Kohany et al. 2006) matched the sequences with both gamma and betaretroviral elements from the database verifying the initial assessment.

**Table 2.** MicrocebusERV genome screening summary.

ERV ID	Blastn hits	Proviruses	soloLTRs	Average LTR evolutionary age estimation	Average proviral evolutionary age estimation	Average gene evolutionary age estimation	Consensus sequence length
MicrocebusERV-1-1	1446	20	348	60 (±28)	54 (±51)	53 (±22)	7104 bp
MicrocebusERV-1-2	2010	109	1627	59 (±58)	50 (±51)	59 (±62)	6811 bp
MicrocebusERV-2-1	457	5	147	44 (±48)	44 (±46)	60 (±56)	7004 bp
MicrocebusERV-2-2	132	39	896	44 (±39	44 (±33)	50 (±29)	5093 bp
MicrocebusERV-2-3	929	138	1102	60 (±56)	58 (±78)	59 (±50)	6701 bp
recMicrocebusERV-1-1	1446	3	N/D	N/D	N/D	N/D	7366 bp

For each provirus, we indicate the contig blastn hits, the number of proviruses, the number of soloLTR sequences identified, and the consensus sequence length. Age estimations of each MicrocebusERV are also shown. N/D indicates not determined.

The exact boundaries for each provirus were annotated and aligned using MAFFT, and recombination analysis using RAT was performed in the multiple sequence alignments of the extracted proviruses identifying a recombinant MicrocebusERV (recMicrocebusERV)-1-1 with MicrocebusERV-2-1 proviral sequences from 5,115 to 6,725 bp. The three recombinant sequences were analyzed separately.

**Figure 2.**
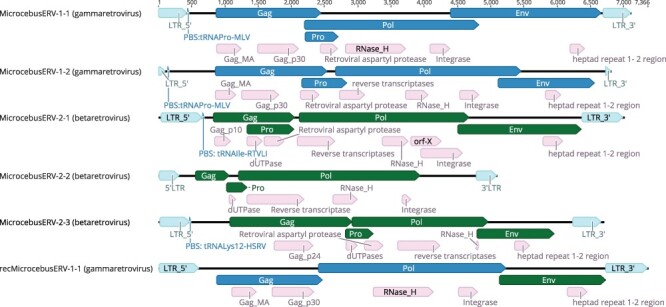
Genomic structure of consensus sequence of identified ERVs. For each ERV consensus sequence, retrotector analysis identified LTRs that are illustrated with light blue, while retrotector identified proviral genes (Gag, Pro, Pol, and Env) that are illustrated in dark blue for the gammaretroviral sequences and in dark green for the betaretroviral sequences. Motifs identified using NCBI CDD for each ERV consensus sequence are illustrated in pink.

Multiple sequence alignments of extracted proviral sequences were also used to generate majority rule consensus sequences for each provirus. The NCBI CDD (Lu et al. 2020) and retrotector (Sperber et al. 2009) were used as independent verification methods to identify the presence of retroviral motifs and genes in the consensus sequences. Retrotector analysis of the gammaretroviral majority rule consensus proviral sequence revealed *gag, protease* (*pro*), *pol*, and *env* genes in both proviral consensus sequences (Sperber et al. 2009). Retrotector analysis of the betaretroviral majority rule consensus proviral analysis was able to identify *gag, protease* (*pro*), and *pol* genes in all the betaretroviruses and *env* gene in MicrocebusERV-2-1 and MicrocebusERV 2–3 (Sperber et al. 2009). Retrotector analysis of the consensus recMicrocebusERV identified a gammaretroviral *pol* gene and betaretroviral *env* gene. A dUTPase domain was identified in all the betaretroviral proviral sequences but, as expected, was not identified in the gammaretroviral proviral sequences. The retrotector script was also able to generate reconstructed retroviral protein (puteins) sequences for the consensus sequence of each provirus (Fig. 2).

A CDD analysis of the majority rule consensus sequences generated from the alignment also identified the same motifs as retrotector for all five proviral sequences and the recombinant sequence. The combined results indicate a high level of conservation of retroviral motifs among the identified proviruses and retroviral proteins.

All the viruses are expressed, as demonstrated by their retrieval from cDNA, and the majority of the generated contigs map to internal parts of the LTRs and genes. While we did not detect hard start and hard stop regions at the start of *env* and the end of *pol*, this may be due to low or inconsistent coverage. The potential open reading frames in the proviruses suggest that multiple different products could be generated. Supplementary Table S1 shows the proviruses that could produce retroviral proteins.

Further analysis of the proviral sequences identified tRNA Pro for both gammaretroviral proviral sequences, tRNA Ile for MicrocebusERV-2-1, tRNA Phe for MicrocebusERV-2-2, and tRNA Lys for MicrocebusERV-2-3 as the tRNAs that would recognize the primer binding site (PBS) for each virus. A PBS was not identified for recMicrocebusERV-1-1. Analysis of the longest and most complete *M. murinus* genome hits of the identified proviruses revealed that the majority of genes are highly mutated and are unlikely to produce fully functional retroviral proteins. However, Supplementary Table S1 shows the proviruses that could potentially produce one or more fully functional retroviral proteins.

## MicrocebusERV age estimation

Age estimation of the identified MicrocebusERV sequences was performed using three different approaches. First, we used a molecular clock approach using the LTR sequences. The identified LTRs for each ERV were used as a template for a blastn search. The results of each search were multiple aligned using MAFFT (Katoh et al. 2005) and manually curated. The resulting curated alignments were used to obtain the Kimura-2-parameter distances of the identified LTR sequences to each LTR majority rule consensus sequence, excluding from the alignment of the CpG dinucleotides positions due to 5-methyl cytosine spontaneous deamination that leads to higher mutation rates in that locations (Kimura 1980). Using the previously published lemur mutation rate of 0.0017/nt/year (Campbell et al. 2021), we estimate the age of each provirus (Table 2). A second dating method used the proviral gene sequences. Each proviral gene consensus sequence was blastn searched in the *M. murinus* genome. Resulting hits were aligned using MAFFT (Katoh et al. 2005) and manually curated. The resulting curated multiple sequence alignments were used to obtain the Kimura-2-parameter distances of the identified gene sequences to each gene majority rule consensus sequence. As with the LTR dating approach, the CpG dinucleotide positions were excluded from the analysis. The age for this approach was calculated using the lemur mutation rate indicated previously. The estimated average age using gene sequences for identified proviruses was calculated (Table 2). The third dating method used the proviral 5ʹ LTR compared to 3ʹ LTR sequences. Retroviruses, upon integration into the host genome, have identical 5ʹ and 3ʹ LTR sequences that acquire mutations independently through time based on the mutation rate of the host. Using the divergence of the two sequences and the *M. murinus* mutation rate, the proviral average age was calculated for each provirus (Table 2).

### Molecular screening of other primates and endemic Madagascar species genomes

Majority rule consensus sequences of each provirus were used for screening a number of primate and Madagascar endemic species genome assemblies obtained from NCBI. Strepsirrhini are estimated to have split from the Haplorrhini ∼87 million years ago (Perelman et al. 2011). Age estimations of the identified proviruses indicate that they should be present in the Strepsirrhini species but not in the Haplorrhini species. A blastn search of the Haplorrhine species failed to produce positive identification for the proviral majority rule consensus sequences. Screening of several Strepsirrhini species genome assemblies revealed positive hits for all five proviral sequences as expected based on the age estimation of the proviral sequences. This included hits in both the Lorisiformes (*O. garnettii* and *N. coucang*) and the Lemuriformes. Table 3 reports the per cent identity for each identified ERV with the *Microcebus* species, the Lemuriformes, and the Lorisiformes, respectively.

**Table 3.** per cent identity between the ERVs and the *Microcebus* species (left column), the ERVs and the Lemuriformes excluding *Microcebus* (middle), and the ERVs and the Lorisiformes (right).

ERV	*Microcebus* spp. (%)	Lemuriformes (%)	Lorisiformes (%)
MicrocebusERV-1-1	91	82	65
MicrocebusERV-1-2	91	85	70
MicrocebusERV-2-1	95	83	67
MicrocebusERV-2-2	89	76	65
MicrocebusERV-2-3	99	81	64

Values are the mean percentage identities from the assemblies screened in each group.

A Blastn search was also performed on genome assemblies of Talazac’s shrew tenrec (*M. talazaci*) and the fossa (*C. ferox*), two other Madagascar endemic species, to examine if the ERVs identified can be found in other isolated species on the island. A gammaretrovirus majority consensus sequences blastn search did not produce positive results with either of the genome assemblies, while the three betaretroviruses yielded positive matches with 70 per cent pairwise identity for *M. talazaci*. Betaretroviruses screening of the *C. ferox* genome assemblies produced a positive hit with 70 per cent pairwise identity in the *gag* gene of MicrocebusERV-2-3. Phylogenetic analysis of the extracted hits from *C. ferox* and *M. talazaci* place them in the same clades as the identified ERVs.

### Phylogenetic analysis of retroviral genomes

**Figure 3.**
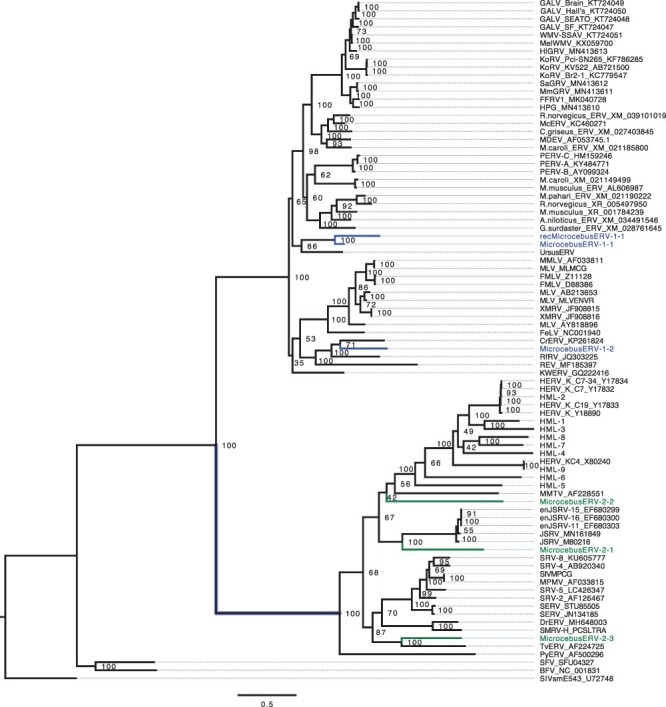
Maximum likelihood phylogenetic analysis of gammaretroviral and betaretroviral majority rule consensus MicrocebusERV sequences compared with NCBI extracted sequences. SIV was used as an outgroup. MicrocebusERV gamma proviral sequences identified are highlighted in blue, while MicrocebusERV beta proviral sequences are in green. MicrocebusERV-1-2 is clustered in the same clade as RfRV and REV, while MicrocebusERV-1-1, recMicrocebus-1-1, and UrsusERV are clustered in the same clade. MicrocebusERV-2-1 lies in the same clade as JSRV sequences, MicrocebusERV-2-2 is in the same clade as Mouse Mammary Tumor virus and HERV-K ERVs, while MicrocebusERV-2-3 is located in the same clade as TvERV.

Retroviral sequences with nucleotide percentage identity >25 per cent to identified mouse lemur ERVs were extracted and aligned using MAFFT. Maximum likelihood phylogenetic analysis was performed on the resulting alignment using SIV as an outgroup. The resulting phylogeny clearly separated the two retroviral genera beta and gamma into two separate nodes (Fig. 3). In the gammaretroviral node, MicrocebusERV-1-2 was placed in the same clade as the RfRV, while the MicrocebusERV-1-1 and recMicrocebusERV-1-1 proviruses were in the same clade as UrsusERV (Tsangaras et al. 2015) (Fig. 3).

In the betaretroviral node, MicrocebusERV-2-1 was placed in the same clade as JSRV sequences, MicrocebusERV-2-2 was in the same clade as Mouse Mammary Tumor virus and HERV-K ERVs, while MicrocebusERV-2-3 clusters with the TvERV (Baillie and Wilkins 2001) (Fig. 3). A maximum likelihood phylogeny of the putein sequence generated from retrotector was also performed. Results of the protein phylogeny were almost identical to the nucleotide sequence analysis, though some clades in the protein-based tree had lower confidence most likely due to insufficient informative sites (Supplementary Figs S2 and S3).

## Discussion

Five ERVs in the *Microcebus* genome, two gammatretroviruses and three betaretroviruses, were identified. All five were found to be present in the Strepsirrhines, including both Lorisiformes and Lemuriformes, but absent from the Haplorrhines, including the tarsier. This suggests that they originated after the Haplorrhine-Strepsirrhine split approximately 87 mya (95 per cent highest posterior density [HPD]: 76–99) but before the Strepsirrhini split into the Lorisiformes and Lemuriformes approximately 69 mya (95 per cent HPD: 59–77) (Perelman et al. 2011).

The estimated ages of these viruses are consistent with the estimated divergence times of the host lineages. Each of the virus age estimates had confidence intervals that fell within the maximum HPD of the Haplorrhine-Strepsirrhine split (99 mya) and the minimum HPD of the lorisiform–lemuriform split: 59 mya) (Perelman et al. 2011). Gammaretroviruses: MicrocebusERV-1-1 was estimated to be 60 (±28) million years old and MicrocebusERV-1-2 to be 59 (±58) million years old; betaretroviruses: MicrocebusERV-2-3 was estimated to be 60 (±56) million years old, MicrocebusERV-2-1 to be 44 (±48) million years old and MicrocebusERV-2-2 to be 44 (±39) million years old.

Moreover, these estimated viral ages are also consistent with evidence that Madagascar was colonized by ancestral lemurs 77–39 mya (Perelman et al. 2011). As would be expected for a virus that originated before the lorisiform–lemuriform split, none of these viruses are dated exclusively after the date range of the colonization. Together, this evidence presents a consistent scenario in which these viruses likely originated in ancestral Strepsirrhines, after the divergence of Haplorrhines, but before the divergence of Lemuriformes and Lorisiformes, i.e. before the colonization of Madagascar.

The betaretroviruses also produced hits in other phylogenetically distant species that are sympatric with lemurs: fossa (*C. ferox*) and tenrecs (*M. talazaci*). The fossa genome produced hits with MicrocebusERV-2-3 and the tenrec genome produced hits with all three betaretroviruses. Carnivora (which includes the fossa) are estimated to have colonized Madagascar between 26 and 19 mya (33–14 mya) and tenrecs between 42 and 25 mya (50–20 mya) (Poux et al. 2005). Because the tenrec confidence intervals overlap with those of both the Carnivora and lemur colonization events, it is possible that tenrecs may have arrived simultaneously, via rafting, with either carnivores or lemurs (Poux et al. 2005). Alternatively, tenrecs may have arrived via a separate rafting event or a land bridge hypothesized to have existed between 45 and 26 mya (Poux et al. 2005; Masters et al. 2021). These colonization dates are broadly consistent with the estimated ages of the betaretroviruses, suggesting that transmission events between the lemurs and the other two taxa may have been possible. One possible scenario is that MicrocebusERV-2-3 was transmitted in Madagascar between lemurs, carnivores that preyed on them, and tenrecs, which were also likely prey. Alternative scenarios include that (1) tenrecs and carnivores may have been independently infected from a common source either before or after colonizing Madagascar, or (2) the primates, fossa, and tenrecs may be carrying closely related but phylogenetically distinct ERVs. For MicrocebusERV-2-1 and Microcebus-2-2, homologs of which were found in the tenrecs but not the fossa, it is not yet clear if and how these species might be linked. Possibilities include that another species may have interacted with both or that the primates and tenrecs may be carrying closely related but phylogenetically distinct ERVs.

It is likely that the proviruses we identified are not intact enough to replicate. While some of the proviruses could potentially produce one or more fully functional proteins (Supplementary Table S1), the majority of genes we detected contained mutations. Although we did not detect *env* in MicrocebusERV-2-2 (Fig. 2), and the loss of *env* functionality can be associated with a switch from transmitting via infection to transmitting via other mechanisms, like retrotransposition (Magiorkinis et al. 2012), we expect that the mutations throughout the other genes render these proviruses incapable of any current replication. Of the two MicrocebusERV-2-2 proviral sequences identified in Supplementary Table S1 as having the potential to code for proteins, one has the potential to code for a *gag* protein and the other for a *pol* protein. Whether the copy numbers observed in our data (ranging from five for MicrocebusERV-2-1 to 138 for MicrocebusERV-2-3) are all the result of reinfections or whether some are due to retrotransposing after losing *env* functionality, but before the subsequent degradation of the other genes, is difficult to determine.

The recombinant virus, recMicrocebus ERV-1-1, contains a gammareretroviral *pol* gene from MicrocebusERV-1-1 and a betaretroviral *env* gene from MicrocebusERV-2-1. This differs from the well-documented pattern in which betaretroviruses acquire gammaretroviral *env* genes, a pattern that has been argued to enable betaretroviruses to expand the range of hosts that they can infect (Henzy and Coffin 2013; Henzy and Johnson 2013). To our knowledge, the evolutionary implications of acquiring a betaretroviral *env* are less well understood and possibly less frequent; however, they do indicate a complex history of retroviral cross-genera recombination.

### The gammaretroviruses

Gammaretroviruses are estimated to have originated early in mammalian evolution and have demonstrated a pattern of frequent intra-order host switching (Hayward, Grabherr, and Jern 2013; Hayward, Cornwallis, and Jern 2015). This pattern appears to also be evident in Strepsirrhines, in that the endogenous gammaretroviruses we detected did not form a clade of their own or with the endogenous gammaretroviruses found in other primates.

#### Microcebus ERV-1-1

MicrocebusERV-1-1 provirus was placed in the same clade with UrsusERV and is estimated to be 60 (±28) million years old. UrsusERV age estimations indicate that the provirus is relatively young and believed to have been circulating in the bear population approximately 10 mya (Tsangaras et al. 2015). Given the large time gap between the origins of MicrocebusERV-1-1 in strepsirrhine primates and the origins of the UmaERV in bears, it is likely that these represent independent germline colonization events by related exogenous retroviruses.

#### Microcebus ERV-1-2

MicrocebusERV-1-2 was dated to about 59 (±58) million years old. It is in the same clade as the RfRV and CrERV. RfRV is basal to most mammalian gammaretroviruses (Cui et al. 2012; Cui, Tachedjian, and Wang 2015). It was detected in the greater horseshoe bat (*R. ferrumequinum*) (Cui et al. 2012), but is thought to have originated in tree shrews (Cui, Tachedjian, and Wang 2015). The similarity of MicrocebusERV-1-2 to RfRV supports the idea that there has been viral transmission among bats, rodents, and primates by related exogenous retroviruses since the beginning of the primate radiation. The basal position of the mouse lemur virus suggests that a hypothesized bat or rodent origin for gammaretroviruses may be premature (Cui et al. 2012; Cui, Tachedjian, and Wang 2015).

CrERV is a gammaretrovirus, which is part of a clade that is found in cervids, including mule deer (*Odocoileus hemionus*), white-tailed deer (*Odocoileus virginianus*), elk (*Cervus canadiensis*), and muntjac (*Muntiacus muntiak*) (Elleder et al. 2012). The presence of this clade of viruses in numerous cervids suggests that the original germline colonization may have occurred before the cervid lineages split approximately 10 mya (Gilbert, Ropiquet, and Hassanin 2006; Elleder et al. 2012). This clade is more distantly related to the endogenous gammaretroviruses of pigs and sheep (Elleder et al. 2012). CrERV, specifically, has been documented to have invaded the mule deer genome when the mule deer and white-tailed deer lineages separated about 1 mya (Elleder et al. 2012). The old age of MicrocebusERV-1-2 and the clustering of viruses from distantly related clades found in distantly related hosts suggest complicated viral transmission patterns that are not yet fully resolved.

### Betaretroviruses

Betaretroviruses are highly abundant in vertebrates, showing strong patterns of intra-class host switching (Hayward, Cornwallis, and Jern 2015). The three endogenous betaretroviruses in the Strepsirrhines are consistent with that pattern in that they do not form a clade together, or with other primates, and suggest that there may have been cross-species transmissions involving fossa and tenrecs. However, we do acknowledge that it is also possible that the betaretroviruses in the Strepsirrhines, fossa, and tenrecs could be closely related, but phylogenetically distinct, viruses.

#### Microcebus ERV-2-1

MicrocebusERV-2-1 is estimated to be 44 (±48) million years old and in the same clade as JSRV. This represents the first non-bovid virus in this viral clade with a 64 per cent identity. Some of the proviral sequences of ERV-2-1 produced a hit for orf-x, an accessory gene with unknown function that overlaps with *pol*, is conserved and thought to be unique to JSRV (Rosati et al. 2000; Griffiths, Martineau, and Cousens 2010; Hofacre and Fan 2010). JSRV has a complex and dynamic evolutionary history and, to our knowledge, is the only known aerosol-transmitted retrovirus. While viruses in the JSRV clade are common in Bovids, older work using Southern blot hybridization suggests that related viruses are also present in other taxa, including some carnivores and primates (Hecht et al. 1996). It is first integrated into the genome of *Ovis* and *Capra* prior to their divergence  5–11 mya (Armezzani et al. 2014; Cumer, Pompanon, and Boyer 2019). Some of the endogenized copies are associated with placental development and may be protective against closely related pathogenic JSRV, making the sheep-JSRV model useful for understanding co-evolutionary dynamics (Armezzani et al. 2014; Cumer, Pompanon, and Boyer 2019). The presence of this virus in Lorisiformes as well as Lemuriformes and its basal position to the JSRVs suggest that the integration time in lemurs is likely to be well before the integration into the germlines of *Ovis* and *Capra* and that this viral group may be more widespread and diverse among wildlife than previously thought.

#### Microcebus ERV-2-2

MicrocebusERV-2-2 was dated at 44 (±39) million years old and was placed in the same clade as Mouse Mammary Tumor virus and HERV-K ERVs. These groups are associated with cancers (Mayer and Meese 2005; Garcia-Montojo et al. 2018); Mouse Mammary Tumor Virus is linked to mammary tumors in mice and the HERV-K superfamily with cancers in humans, as well as neurodegenerative diseases (Mayer and Meese 2005; Garcia-Montojo et al. 2018). HERV-K viruses are well documented in Catarrhines, including humans (Mayer and Meese 2005; Garcia-Montojo et al. 2018), and some are reported in Platyrrhini (Kim et al. 1999; Lavie et al. 2004). Given the frequency of host switching in betaretroviruses (Hayward, Cornwallis, and Jern 2015), it is not possible to determine whether these germline colonization events, or the colonization of the tenrec germline, have a single origin.

#### Microcebus ERV-2-3

MicrocebusERV-2-3 was estimated to be 60 (±56) million years old. It is basal to the other four identified retroviral clades (Fig. 3). As ERV-2-3 is 97.1 per cent similar to AC145758, it is very likely to be the same endogenous *M. murinus* betaretrovirus previously identified in a BAC sequence (Baillie et al. 2004). The virus is basal in the phylogenetic tree and appears to be in the same clade as TvERV, an endogenous type D betaretrovirus found in the common brushtail opossum, *T. vulpecula*, and retrovirus elements in the mouse and rat genomes. MicrocebusERV-2-3 and TvERV also formed a clade with the SRV ((Baillie and Wilkins 2001) and Fig. 3), with SMRV-H and DrERV (from vampire bats in Mexico). Thus, this clade includes viruses infecting hosts which are both phylogenetically and geographically distant, suggesting a complex and still largely unknown history of cross-species transmission.

## Conclusion

This study indicates that Strepsirrhines have been evolving and experiencing retroviral genome colonization events since before the lorisiform–lemuriform split. That the mouse lemur viruses largely cluster with viruses in non-lemur, and indeed, non-primate hosts, suggests that patterns of frequent host switching by gamma- and betaretroviruses have also occurred in these lineages. Much of the diversification of ERVs in mouse lemurs took place before the colonization of Madagascar, though further infection of other species in Madagascar, such as tenrecs and fossas, could potentially have occurred subsequently. Some of the discovered phylogenetic associations suggest interspecies transmission is both temporally and spatially complex, e.g. the squirrel monkey- and vampire bat-like MicrocebusERV-2-3, or suggests the origins of known clades may be far older than previously estimated, e.g. the JSRV-like MicrocebusERV-2-1. Further sequencing of wildlife will likely help identify and resolve the associations of many additional retroviral clades.

## Supplementary Material

veac117_Supp

## Data Availability

SRA Accession ID: PRJNA779192.
