# Supplementary material for: Long-term host–pathogen evolution of endogenous beta- and gammaretroviruses in mouse lemurs with little evidence of recent retroviral introgression
Source: Virus Evol. 2022 Dec 14;9(1):veac117. doi: 10.1093/ve/veac117 (PMC9825726; doi:10.1093/ve/veac117)

**Supplementary Tables**

**Supplementary Table 1.** MicrocebusERV sequences with coding potential.


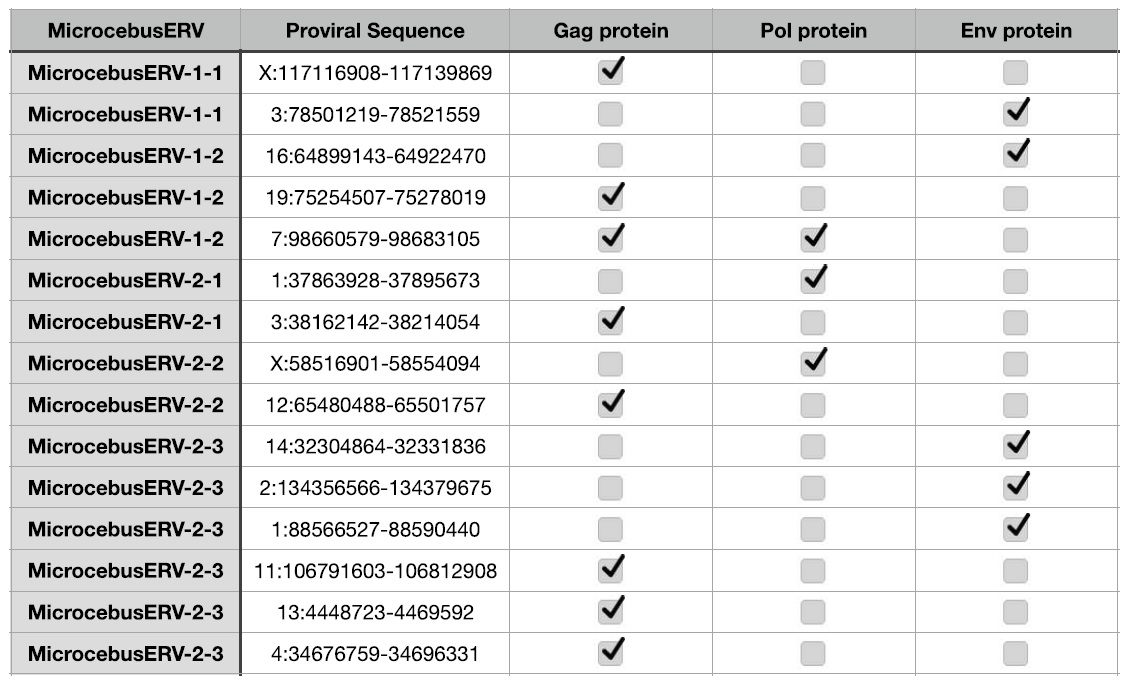


**Supplementary Figures**

**Supplementary Figure 1:** Alignment of cDNA constructed contigs that were used for retrieval of full length proviruses to MicrocebusERV consensus sequences. **A**) Alignment of MicrocebusERV-1-1 generated contigs, **B**) Alignment of MicrocebusERV-1-2 generated contigs, **C**) Alignment of MicrocebusERV-2-1 generated contigs, **D**) Alignment of MicrocebusERV-2-2 generated contigs, **E**) Alignment of MicrocebusERV-2-3 generated contigs.

**
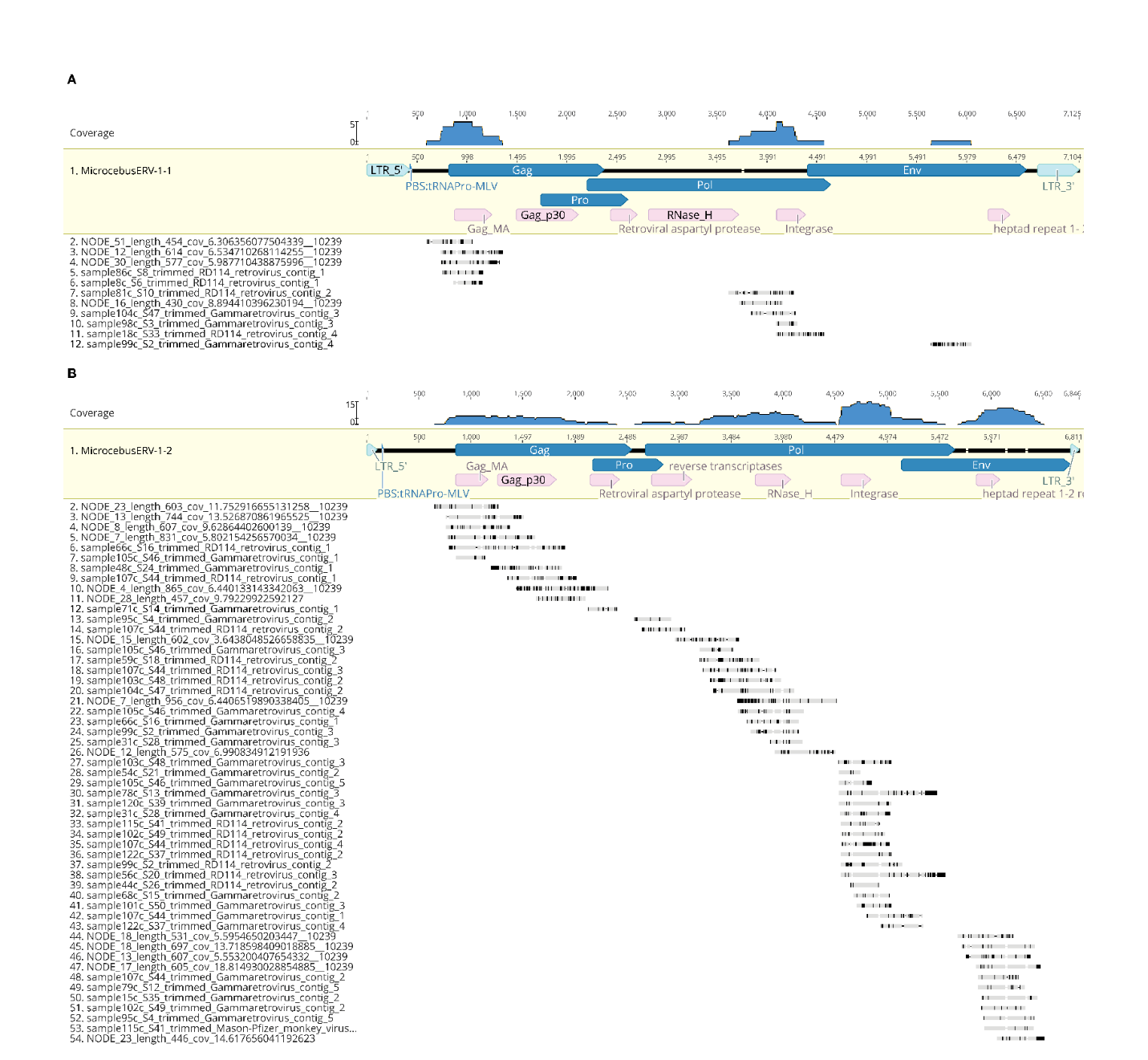
**

**
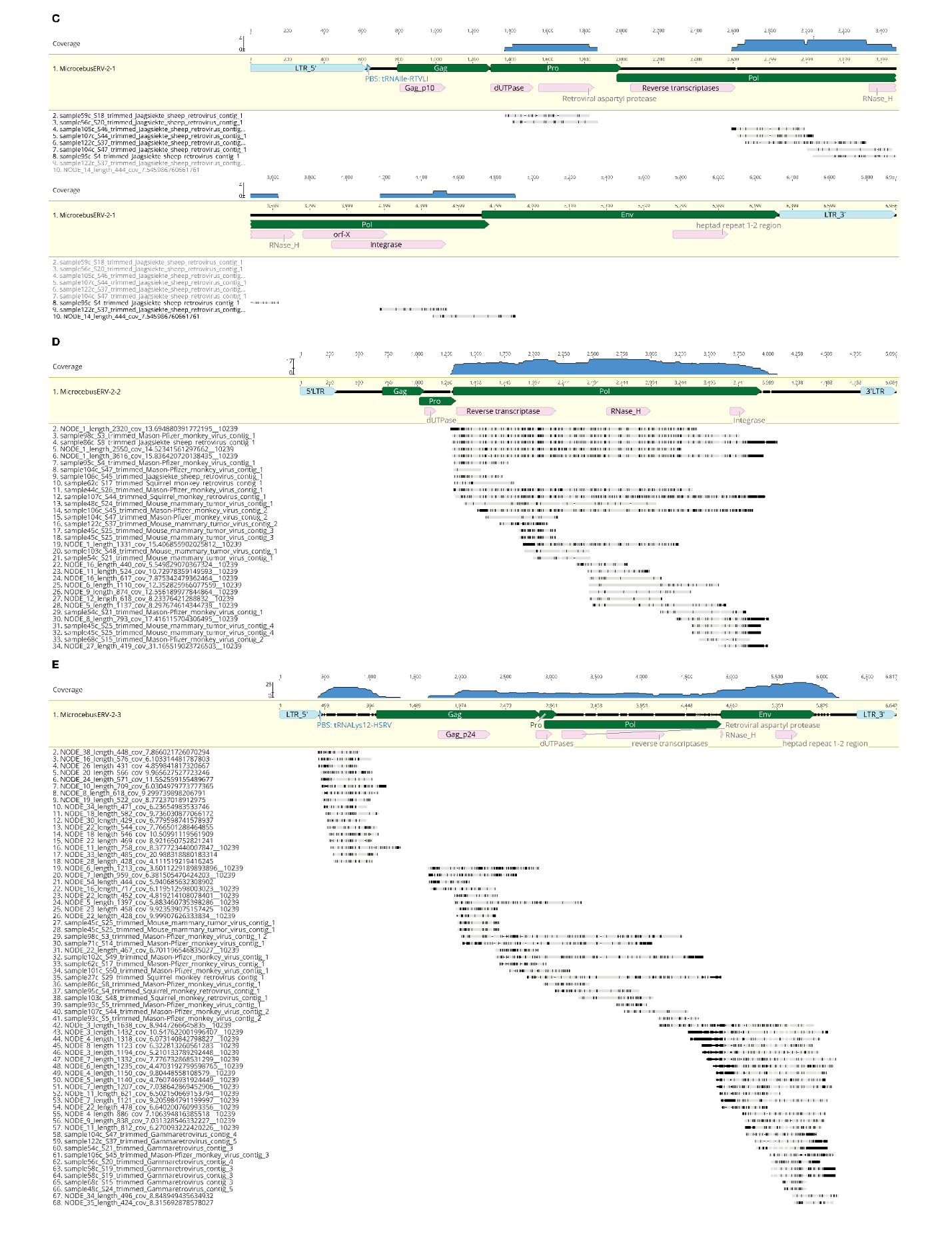
**

**Supplementary Figure 2**: Putein trees generated with RAxML for the gammaretroviral genes of each identified gammaretrovirus. A: *gag*, B: *pol*, C: *env*.


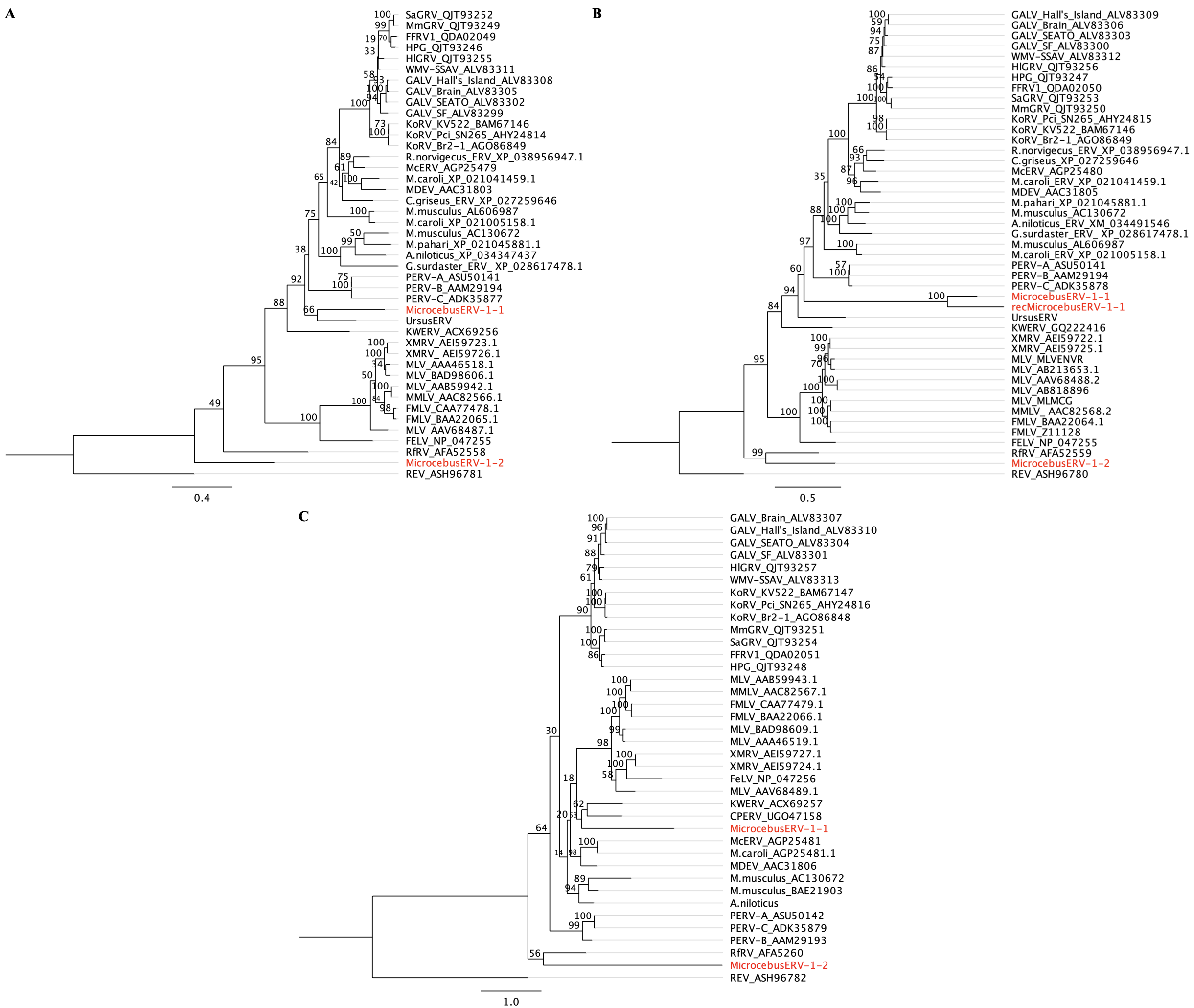


**Supplementary Figure 3**: Putein trees for each gene generated with RAxML for the identified betaretroviruses. A: *gag*, B: *pro*, C: *pol,* D*: env*.


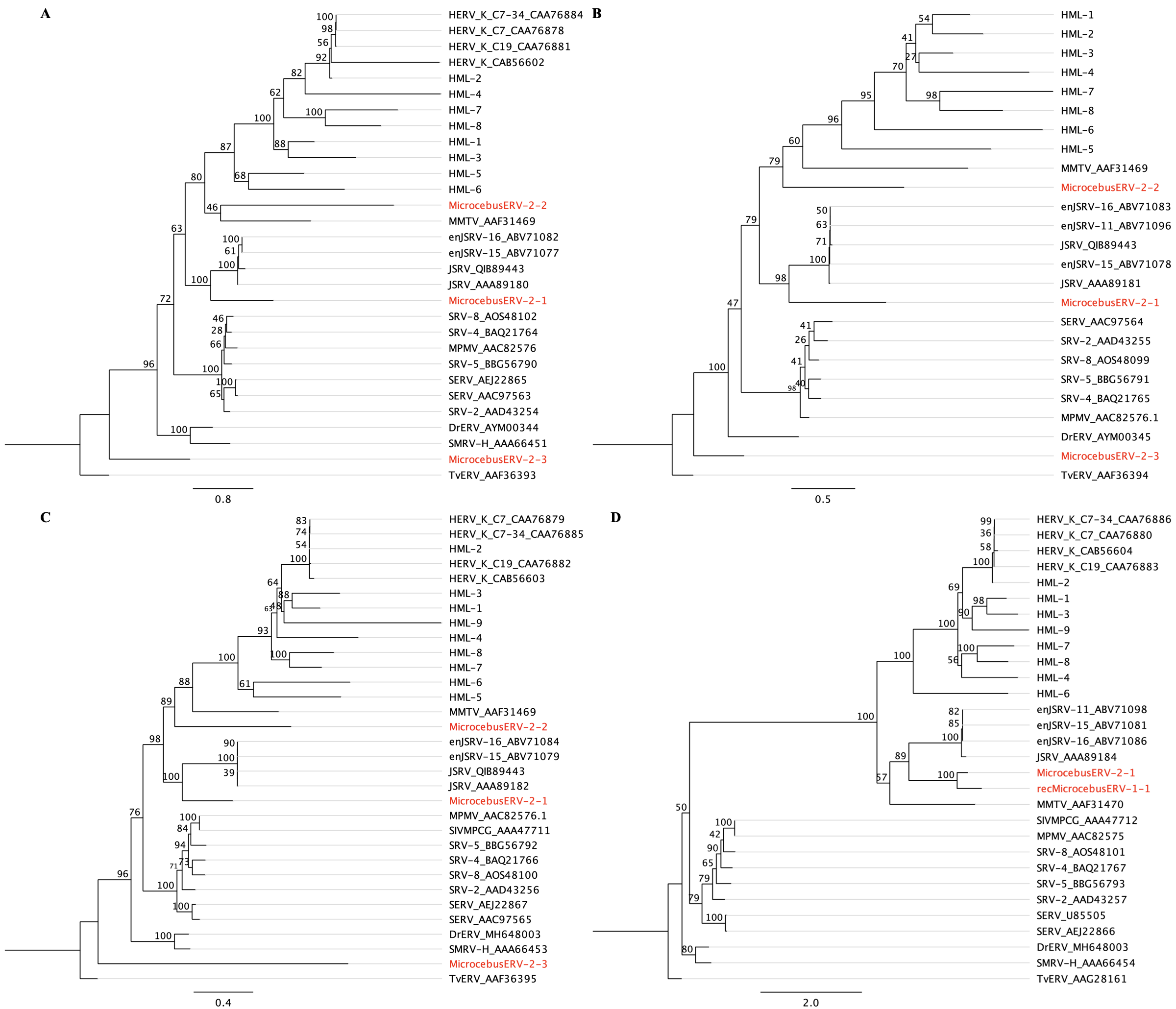

Supplement: veac117_Supp [file veac117_supp.zip › suppl_data/Supplementary_File_with_FIGs_Fig1_FULL.docx]
